# A novel homozygous nonsense mutation in NECTIN4 gene in a Pakistani family with ectodermal dysplasia syndactyly syndrome 1^⋆^

**DOI:** 10.1016/j.abd.2022.07.009

**Published:** 2023-05-12

**Authors:** Bibi Hajra, Abdullah, Nousheen Bibi, Fibhaa Syed, Asmat Ullah, Wasim Ahmad, Umm-e-Kalsoom

**Affiliations:** a Department of Biochemistry, Faculty of Biological and Health Sciences, Hazara University, Mansehra, KP, Pakistan; b Department of Biochemistry, Faculty of Biological Sciences, Quaid-i-Azam University, Islamabad, Pakistan; c Department of Bioinformatics, Shaheed Benazir Bhutto Women University, Peshawar, KP, Pakistan; d Department of General Medicine, Shaheed Zulfiqar Ali Bhutto Medical University, PIMS, Islamabad, Pakistan; e Novo Nordisk Foundation Center for Basic Metabolic Research, Section of Metabolic Genetics, Faculty of Health and Medical Sciences, University of Copenhagen, Copenhagen, Denmark

**Keywords:** Ectodermal dysplasia, Palmoplantar keratoderma, Poliovirus Receptor Related-4, Nectin cell adhesion molecule-4, Ectodermal dysplasia syndactyly syndrome 1

## Abstract

**Background:**

Ectodermal dysplasia syndactyly syndrome 1 (EDSS1) is a rare hereditary disorder characterized by defects in teeth, hair, and nails in association with a fusion of the digits. Genetically, the disease phenotypes are caused by homozygous and compound heterozygous variants in NECTIN4 gene.

**Objective:**

The main objective of the study was to identify the pathogenic sequence variant(s) for family screening and identification of carriers.

**Methods:**

In the present study, the authors have investigated a large consanguineous family of Pakistani origin segregating autosomal recessive EDSS1. All the coding exons of the NECTIN4 gene were directly sequenced using gene-specific primers.

**Results:**

The affected individuals presented the classical EDSS1 clinical features including sparse hair, hypoplastic nails with thick flat discolored nail plates, peg-shaped, conical, and widely spaced teeth with enamel hypoplasia, proximal cutaneous syndactyly of fingers and toes. Sequence analysis of the coding region of the NECTIN4 identified a novel nonsense variant [c.163C>T; p.(Arg55*)] in exon-2 of the gene. Computational analysis of protein structure revealed that the variant induced premature termination at Arg55 located in Ig-like V-loop region leading to loss of Ig-C2 type domains and transmembrane region, and most likely Nectin-4 function will be lost.

**Study limitation:**

Gene expression studies are absent that would have strengthened the findings of computational analysis.

**Conclusion:**

The present study expanded the phenotypic and mutation spectrum of the NECTIN4 gene. Further, the study would assist in carrier testing and prenatal diagnosis of the affected families.

## Introduction

Ectodermal Dysplasias (EDs) constitute a group of congenital disorders affecting the skin and its appendages including hair, teeth, nails, and sweat glands. EDs are heterogeneous both in genetic causes and clinical phenotypes with more than 200 different forms reported to date and an estimated prevalence of approximately 7/10,000 births globally.^1^ Initial classification systems were based mainly on the phenotypic features and mode of inheritance. More recent approaches include a variety of molecular data that facilitate establishing the relationship of genetic defect with the effect on protein structure and function and the resultant phenotypes.^2^

ED involving congenital hair abnormalities along with cutaneous syndactyly is a very rare form that has been mapped on chromosomes 1q23.1-q23.3 and 7p21.1-p14.3 causing ED Syndactyly Syndrome-1 (EDSS1; OMIM 613573) and EDSS2 (OMIM 613576) respectively. The characteristic clinical features include sparse to absent scalp hair, sparse eyebrows, and eyelashes, abnormal dentition (peg-shaped, conical crowns and enamel defects), hypoplastic nails, palmoplantar keratoderma and bilateral partial cutaneous syndactyly variably affecting the fingers and toes.^3^, ^4^ EDSS1 has been reported to be caused by variants in the Poliovirus Receptor Related-4 (PVRL4) gene recently named Nectin Cell Adhesion Molecule-4 (NECTIN4). NECTIN4 encodes a member of the Nectin family of cell adhesion molecules, Nectin-4 that shows high expression in Adheren Junctions (AJ) of the suprabasal epidermis, hair follicles, cultured keratinocytes and in separating digits of the murine embryo.^4^ The causative gene for EDSS2 has not been reported yet.

Here, the authors report a large Pakistani family with fifteen affected individuals segregating autosomal recessive EDSS1. All the affected individuals presented hair and teeth abnormalities along with bilateral syndactyly. Direct sequencing of the candidate gene identified a novel homozygous nonsense variant [c.163C>T; p.(Arg55*)] in the NECTIN4 gene thus expanding the phenotypic and molecular spectrum of this rare entity.

## Methods

The study was conducted in accordance with the Helsinki Declaration of 2013. Informed written consent was obtained from study participants and approval was obtained from Institutional Review Board and ethical review committee (F. nº 73/HU/ORIC/2021/754) of the parent institution.

### Pedigree drawing and DNA extraction

**Figure 1:**
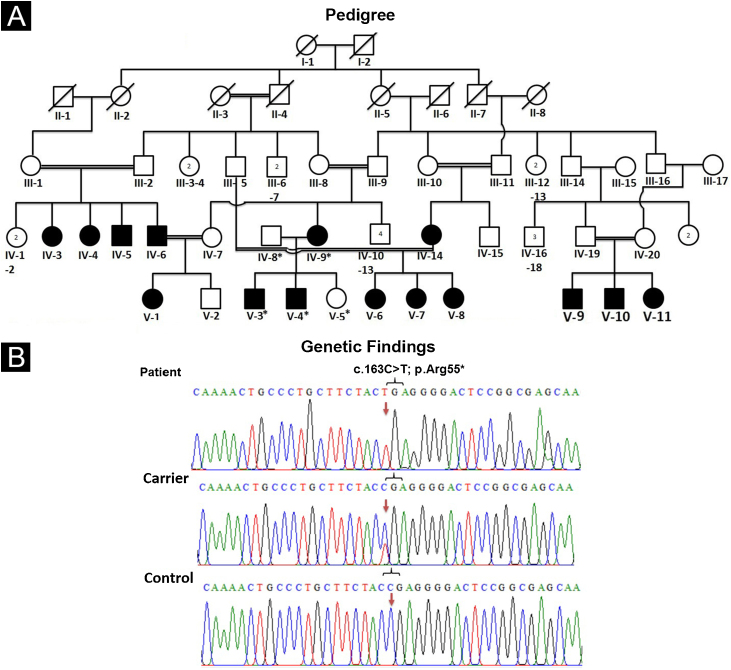
(A) Pedigree of an affected family showing segregation of EDSS1 in autosomal recessive form. (B) Sequencing chromatogram of NECTIN4 gene indicating nonsense variant (c.163C>T) in homozygous state in affected individuals (upper panel), in heterozygous state in carrier (IV-8) (middle panel) and homozygous wild type (C) allele in normal (lower panel). Arrows are used to highlight the position of variation

For pedigree construction (Fig. 1A), blood collection, and clinical diagnosis, the affected members (IV-9, V-3, V-4) were visited at their residence in the Mansehra district, Hazara division, Khyber Pakhtunkhwa, Pakistan. All the participating individuals were briefed about the purpose and the consequences of the research project. Well-informed elders of the family were interviewed for pedigree drawing and disease onset. Venous blood samples were drawn from an antecubital vein. The collected blood was stored in EDTA-containing tubes labeled with individuals’ IDs. Genomic DNA was extracted from all the collected blood samples using a standard phenol-chloroform procedure. DNA was quantified using a Nanodrop-1000 spectrophotometer (Thermal Scientific, Wilmington, MA).

### Targeted gene sequencing

Based upon the disease inheritance pattern, consanguinity, and association of NECTIN4 gene with similar disease phenotypes targeted Sanger sequencing was performed using the DNA of an affected Individual (IV-9). Primers for sequencing the coding exons and intron-exon boundaries of NECTIN4 were designed to form intronic regions using PRIMER-3 software (http://frodo.wi.mit.edu/primer3). Specificity of primers was checked using a basic local alignment search tool (BLAST; http://www.ncbi.nlm.nih.gov/blast). Nucleotide sequences of the primers are available on request. Primers were amplified, purified, and sequenced using standard conditions.^5^ PCR-amplified products were purified using a commercially available kit (Axygen, CA, USA). Sanger sequencing was performed using a Big Dye Terminator v3.1 Cycle Sequencing Kit (Life Technologies, Carlsbad, CA, USA). BIOEDIT sequence alignment editor, version 6.0.7 (Ibis Biosciences, Carlsbad, CA, USA) was used for variants identification. MutationTaster (http://www.mutationtaster.org/) was used for calculating the pathogenicity score of identified variant.

### Secondary and tertiary structure of Nectin-4

The crystal structure of the human Nectin-4 extracellular fragment was retrieved from Protein Data Bank (PDB) (https://www.rcsb.org/) with PDBID:4FRW. The crystal structure is available in dimer conformation encompassing the D1-D2 extracellular domain of Nectin-4. Chimera 1.5.6,^6^ and VEGA ZZ (http://www.ddl.unimi.it) were used for energy minimization and structure refinements. The secondary structure was retrieved through PDBsum (http://www.ebi.ac.uk).

## Results

### Clinical findings

**Figure 2:**
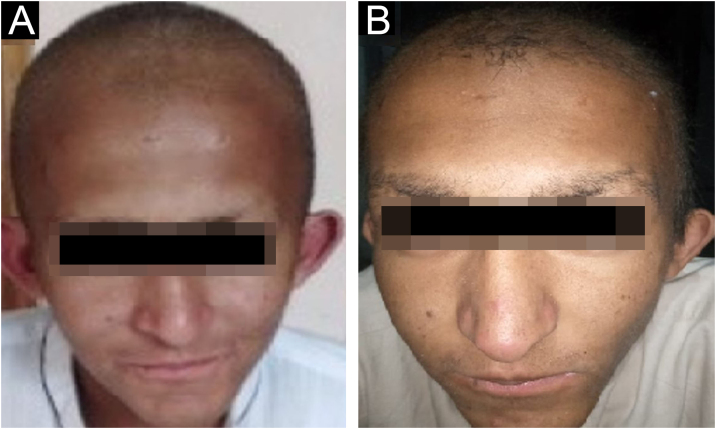
Facial photographs of affected individuals (V-3) and (V-4) are shown in panel A and B respectively. Sparse scalp hair, sparse eyebrows and eyelashes and deformed pinnae of both ears are clearly evident

**Figure 3:**
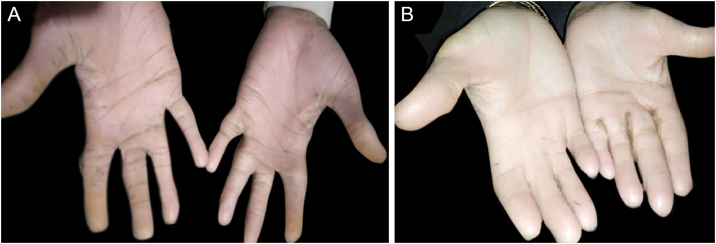
Hands of affected member (V-4) and (IV-9) showing large palm size, short fingers, dry scaly skin, keratoderma, and proximal cutaneous syndactyly of 2^nd^, 3^rd^ and 4^th^ fingers in V-4 (A) and in right hand of IV-9 (B)

**Figure 4:**
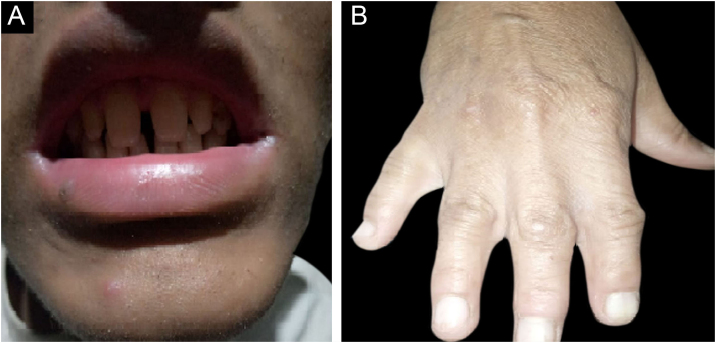
(A) Peg-shaped, conical, and widely spaced teeth with enamel hypoplasia. (B) Hand of an affected member (IV-9) showing hypoplastic, discolored nail plate

**Figure 5:**
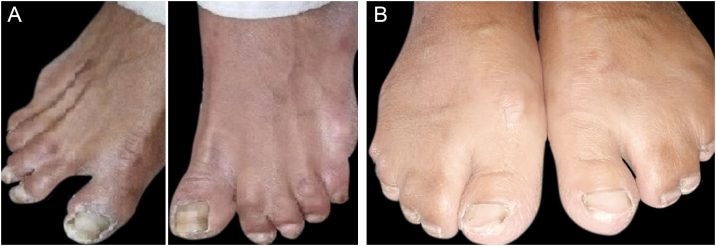
(A) Feet of an affected member (V-3) showing bilateral 2‒3 toes syndactyly (B) Feet of an affected mother (IV-9) showing fusion of 2^nd^ to 5^th^ toes up to distal interphalangeal joint

All the affected individuals in the family showed EDSS1-specific ectodermal phenotypes i.e. abnormal hair, teeth, nails, and skin. Hair phenotypes observed in affected individuals included sparse scalp hair, sparse eyebrows, and eyelashes while the hair on the rest of the body was absent (Fig. 2). Their skin was dry and scaly with hyperkeratosis and palmoplantar keratoderma (Fig. 3). The teeth were peg-shaped, conical, and widely spaced with enamel hypoplasia (Fig. 4A). All the affected members had large palm sizes, and short fingers (Fig. 2). The nails were hypoplastic with a discolored nail plate (Fig. 4B). The proximal cutaneous syndactyly of 2^nd^, 3^rd^ and 4^th^ fingers was observed bilaterally in two affected members (V-3, V-4) (Fig. 3A) and in the right hand of an affected mother (IV-9) (Fig. 3B). Similarly, syndactyly of 2^nd^ and 3^rd^ toe was observed in feet of V-3 and V-4 (Fig. 5A) while 2^nd^ to 5^th^ toes of the mother (IV-9) were fused cutaneously up to a distal interphalangeal joint (Fig. 5B). Additionally, they had almost negligible sweating and faced the problem of heat intolerance during summer.

### Genetic findings

The pedigree analysis clearly indicated the autosomal recessive inheritance pattern. Comparison of sequencing results generated from patients’ (IV-9, V-3, V-4) DNA with reference sequence revealed a homozygous sequence variant (c.163C>T) in exon-2 of the gene (Fig. 1B). Sequencing of the identified variant in normal members (IV-8, V-5) confirmed its heterozygous state, consistent with the autosomal recessive mode of inheritance. The variant was not present in 50 ethnically matched healthy individuals. Pathogenicity of the variant complied with ACMG guidelines^7^ and consistent clinical phenotypes. The variant was not present in ExAC or 1000G and was predicted as disease-causing by Mutation Taster.

### Structural analysis of Nectin-4

**Figure 6:**
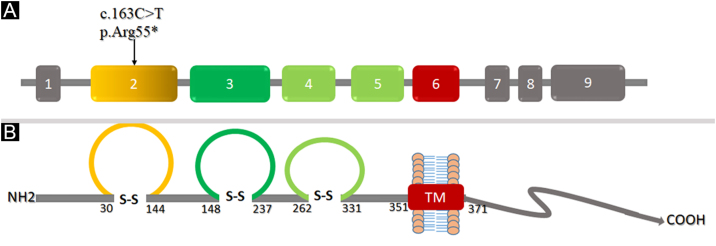
(A‒B) Domain architecture of Nectin-4 showing three extracellular Ig-like domains in yellow and green color respectively while transmembrane region in shown in red color. The numbers denote the amino acids located at the boundaries of each domain

**Figure 7:**
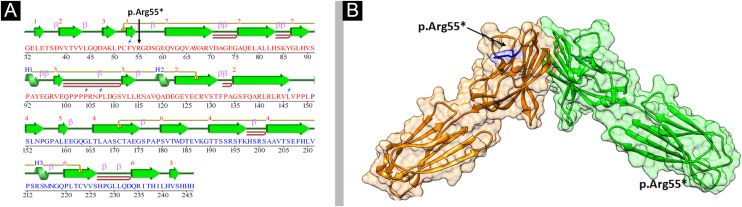
(A) Secondary structure of Nectin-4 D1-D2 domain. (B) 3D structure of Nectin-4 dimer in the range of 32‒243 amino acid. Two subunits of dimer are represented in orange and green ribbon with Arg55 shown in blue stick model

To understand the conformational changes in the three-dimensional structure of a protein, knowledge of secondary structure features is essential. The identified variant (p.Arg55*) was mapped in the first Ig-V-like domain of Nectin-4 encompassing 30‒144 residues (Fig. 6). 2D structure analysis revealed 6 β-sheets, 3 α-helices, and 2 disulfide bridges (Fig. 7A). Termination of Nectin-4 at Arg55 position resulted in the loss of all secondary structure and ultimately loss of tertiary structure and its biological interactions.

**Figure 8:**
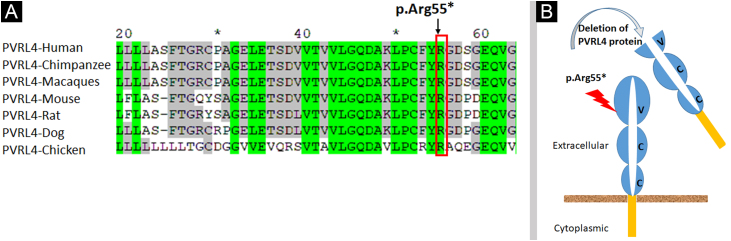
(A) Sequence alignment showing conservation of Arg55 among seven species. (B) Schematic model representing deletion of Nectin-4 protein functional domains upon Arg55* variant

Analysis of the 3D model of Nectin-4 D1-D2 extracellular domain indicated that Arg55 is located in an Ig-like V-loop region that is critical for making cis and trans dimers of Nectin-4 (Fig. 7B) and this is highly conserved among different species (Fig. 8A). Due to premature termination, the Nectin-4 protein will lose both Ig-C2 type domains and transmembrane region (Fig. 8B), most likely the loss of Nectin-4 function that led to the EDSS1 phenotypes.

## Discussion

The Nectins comprising four members (Nectin 1‒4) constitute a family of cell adhesion molecules that belong to the Immunoglobulin Superfamily (IgSF). These proteins either independently or along with other cadherin proteins play a significant role in adhering junctions formation that mediates cell-cell adhesion.^8^, ^9^, ^10^, ^11^ All the Nectins have a characteristic structure comprising of an ectodomain made up of three Immunoglobulin (Ig)-like domains, a single transmembrane region, and a cytoplasmic domain.^12^ The cytoplasmic domain recognizes an Afadin, the adaptor molecule that binds and recruits Filamentous actin assemblies (F-actin), which facilitate cell–cell adhesion.^13^, ^14^ The Ig-like domains of nectins are specialized in mediating hemophilic or heterophilic interactions. Particularly, nectin-1 and nectin-4 trans-interactions regulate the reformation of the actin cytoskeleton through the activation of Rac1, a member of the Rho family of small GTPases that enhances the clustering of adhesion molecules.^12^, ^15^ Defective trans-dimerization due to loss or impairment of either Nectin-1 (also known as PVRL1) or Nectin-4 causes a defect in normal cell-cell adhesion and causes Cleft Lip/Palate ED (CLPED1) and EDSS1 respectively^4^, ^16^, ^17^ both characterized by abnormalities in hair and teeth along with cutaneous syndactyly.

Sanger sequencing of the coding exons of the NECTIN4 gene identified a novel homozygous nonsense variant (c.163C>T) in the affected individuals of the family reported here. This variant is predicted to result in loss of Nectin-4 function either via Nonsense-Mediated mRNA Decay (NMD) or through the production of truncated protein. Computational analysis of 2D and 3D protein structures confirmed that this variant (p.Arg55*) caused premature termination of Nectin-4 hence major part of the Ig domain, a transmembrane domain, and cytoplasmic domain will not be encoded. Thus, the function of the Nectin-4 protein (cell-cell adhesion) is impaired/lost in RAC1 signaling pathway leading to the disease phenotypes of EDSS1.

This is the third nonsense variant in the NECTIN4 gene thus bringing the total number of variants to eleven. Previously five missense (p.Leu81Pro, p.His83Tyr, p.Pro212Arg, p.Val242Met, p.Arg284Gln), a frameshift (p.Gln384ArgfsTer7), Exon 2 in-frame deletion, two nonsense (p.Asp61Ter, p.Gln77Ter) and a compound heterozygous variant (p.Thr185Met; p.Pro304HisfsTer2) have been reported in NECTIN4 gene.^4^, ^18^, ^19^, ^20^, ^21^, ^22^, ^23^, ^24^, ^25^ Most of the clinical phenotypes of the patients in the present study like cutaneous syndactyly of 2-3-4 fingers and 2-3-4-5 toes, thick flat discolored nail plate with hyperkeratosis of finger and toenails, palmoplantar keratoderma, deformed pinnae, negligible sweating, and heat intolerance were also reported by Raza et al. ^21^ This is most probably due to the reason that both the variants are nonsense and are located in the Ig-like V-loop region that is critical for making cis and trans dimers of Nectin-4. As compared to the present study, the enamel hypoplasia was most severe with ill-defined crown surface morphology in a Kashmiri family inheriting nonsense (p.Asp61*) variant. Our patients had sparse short hair on the scalp, sparse eyebrows, and eyelashes as reported previously^19^, ^20^, ^21^, ^22^, ^23^, ^24^, ^25^ but the hair anomaly was congenital and not progressive as reported in families described by Brancati et al.;^4^ Fortugno et al.^24^ and Florian et al.^25^

All the sequence variants reported to date in NECTIN4 in Pakistani families and Pakistani-administered Kashmir families segregating EDSS1 are present in the extracellular domain of Nectin-4. The variant (p.Arg55*) identified in the present study is also located in the extracellular domain. Therefore, it is suggested that families of the same origin afflicted with EDSS1 may be checked for variations in the region encoding extracellular domain before performing whole genome/exome sequencing.

## Conclusion

The study not only expanded the spectrum of mutations in the NECTIN4 gene but also emphasizes the importance of targeted gene testing as a powerful diagnostic tool for a conclusive determination of the specific type of ED.

## Financial support

None declared.

## Authors' contributions

Bibi Hajra: Data collection, analysis, and interpretation.

Abdullah Abdullah: Critical literature review; effective participation in research orientation.

Nousheen Bibi: Data collection, analysis, and interpretation.

Fibhaa Syed: Data collection, analysis, and interpretation.

Asmat Ullah: Preparation and writing of the manuscript.

Wasim Ahmad: Approval of the final version of the manuscript; manuscript critical review.

Umm-e-Kalsoom: Preparation and writing of the manuscript, study conception and planning.

## Conflicts of interest

None declared.
